# Preventing Rejection of the Kidney Transplant

**DOI:** 10.3390/jcm12185938

**Published:** 2023-09-13

**Authors:** Divyanshu Malhotra, Priyanka Jethwani

**Affiliations:** 1 Johns Hopkins Medicine, Johns Hopkins Comprehensive Transplant Center, Baltimore, MD 21287, USA; 2 Methodist Transplant Institute, University of Tennessee Health Science Center, Knoxville, TN 37996, USA; pjethwan@uthsc.edu

**Keywords:** preventing rejection, HLA matching, immunosuppression, biomarkers, emerging therapies, desensitization

## Abstract

With increasing knowledge of immunologic factors and with the advent of potent immunosuppressive agents, the last several decades have seen significantly improved kidney allograft survival. However, despite overall improved short to medium-term allograft survival, long-term allograft outcomes remain unsatisfactory. A large body of literature implicates acute and chronic rejection as independent risk factors for graft loss. In this article, we review measures taken at various stages in the kidney transplant process to minimize the risk of rejection. In the pre-transplant phase, it is imperative to minimize the risk of sensitization, aim for better HLA matching including eplet matching and use desensitization in carefully selected high-risk patients. The peri-transplant phase involves strategies to minimize cold ischemia times, individualize induction immunosuppression and make all efforts for better HLA matching. In the post-transplant phase, the focus should move towards individualizing maintenance immunosuppression and using innovative strategies to increase compliance. Acute rejection episodes are risk factors for significant graft injury and development of chronic rejection thus one should strive for early detection and aggressive treatment. Monitoring for DSA development, especially in high-risk populations, should be made part of transplant follow-up protocols. A host of new biomarkers are now commercially available, and these should be used for early detection of rejection, immunosuppression modulation, prevention of unnecessary biopsies and monitoring response to rejection treatment. There is a strong push needed for the development of new drugs, especially for the management of chronic or resistant rejections, to prolong graft survival. Prevention of rejection is key for the longevity of kidney allografts. This requires a multipronged approach and significant effort on the part of the recipients and transplant centers.

## 1. Introduction

Our understanding of the role of the immune system in allograft survival has evolved immensely since the first human-to-human kidney transplant in 1933 which failed promptly due to acute rejection [1]. With increasing knowledge of immunologic factors and with the advent of potent immunosuppressive agents, the last several decades have seen significantly improved allograft survival. Today, median graft survival ranges from 11.7 years in deceased donor kidney transplants up to 19.2 years in living donor kidney transplants [2]. However, despite overall improved short to medium-term allograft survival, long-term allograft outcomes remain unsatisfactory [3].

A large body of literature implicates acute rejection as an independent risk factor for graft loss [4,5,6,7,8,9,10]. In addition, patients who develop acute rejection early after transplant are at higher risk of chronic allograft rejection [11,12]. With the growing burden of kidney disease and a limited organ pool, it is imperative to understand the factors contributing to allograft rejection to maximize allograft longevity. Preventing rejection in the kidney allograft is a fundamental process to minimize the risk of graft loss, prolong graft survival and decrease the need for organs required for re-transplantation, which limits the organ pool further. Thus, in this article, we review measures taken at various stages in the kidney transplant process to minimize the risk of rejection [Table 1].

## 2. Pre-Transplant Measures

### 2.1. Optimization of Donor-Recipient Compatibility: HLA Matching

The human leukocyte antigen (HLA) system is a complex set of highly polymorphic antigens that are responsible for immune activation by antigen presentation and recognition of ‘self’ and ‘non-self’. Although >25,000 HLA alleles have been identified, only 60 to 70 of the most frequently occurring alleles are considered when determining compatibility for organ transplantation [13]. Mismatches in donor and recipient HLA may lead to activation of alloreactive T-cells and the production of antibodies against the allograft (DSAs) thereby limiting survival. Minimizing HLA mismatches is, therefore, one of the cornerstones of reducing allograft immunogenicity.

The benefit of better HLA matching in reducing acute rejection risk has been repeatedly demonstrated. Compared to a 0 HLA mismatch, any increase in total burden of HLA mismatches at the HLA-A, B, DR loci is associated with an incremental rate of rejection [14] and overall worse allograft survival [15]. There are now also convincing data that, in addition to HLA-ABDR mismatches, mismatches at DQ are independently associated with acute rejection, regardless of initial immunosuppression with a HR of 2.85 (95% CI, 1.05 to 7.75) when there is a two-allele mismatch [16]. The conventional HLA mismatch approach is now also being challenged by more precise analyses of HLA epitopes, which are the specific antigen binding sites of the HLA molecule and eplets, which are clusters of amino acids on the antibody biding sites [17]. There is increasing evidence that eplet mismatches rather than broad HLA mismatches alone are associated with acute rejection in kidney transplant recipients. Senev et al. observed that eplet mismatch load was associated with increased rate of de novo DSA occurrence and graft failure, especially at the DQ antigen with the odds for T cell- or antibody-mediated rejection increasing by 5% and 12%, respectively, per mismatch [18]. In another study, even within recipients who were considered low immunological risk (0–2 broad antigen HLA-ABDR mismatch), those with 20 or greater eplet mismatches experienced an increased risk of rejection compared to those with fewer than 20 mismatches (adjusted HR, 1.85; 95% CI, 1.11–3.08; *p* = 0.019) [19]. As such, algorithms have been developed for more precise identification of amino acids on immunogenic HLA molecules [20] to predict rejection risk and tailor immunosuppression to prevent rejection.

For deceased donor organ recipients, while the degree of HLA mismatch is not modifiable, this information can certainly guide induction immunosuppression therapy (discussed later), as well as allow the individualization of maintenance immunosuppression to minimize kidney transplant rejection. However, in the scenario of a living donation where there are multiple potential donors, the donor with the lowest antigen mismatch load may be selected preferentially. Transplant centers are increasingly enrolling HLA compatible living donor pairs in the kidney-paired donation program. A multi-center study demonstrated that 26.9% of otherwise compatible pairs enrolled in such a program and more than half successfully received an equivalent or better match [21]. Several groups have created simulations to demonstrate that HLA compatible pairs enrolled may be able to achieve significantly lower HLA class I and class II total and antibody-verified eplet mismatch load through the paired kidney donation program [22].

### 2.2. Desensitization in HLA-Incompatible Kidney Transplantation

Approximately 20% of the kidney transplant recipient pool is ‘sensitized’, which refers to the presence of antibodies against commonly found HLA antigens that may be donor-specific and may render the donor and recipient HLA-incompatible. Common reasons for HLA antibody development include prior blood transfusions, multiple pregnancies and prior organ transplantation [23]. Presence of even low level DSAs prior to transplantation can nearly double the risk of antibody-mediated rejection (RR 1.98; 95% CI 1.36–2.89; *p* < 0.001) and increase the risk for graft failure (RR, 1.76; 95% CI, 1.13–2.74; *p* = 0.01) [24].

However, in the case of highly sensitized patients (cPRA > 98%) that have a potential living donor or have been waiting for a deceased donor transplant for a long time, some centers opt for desensitization to improve the likelihood of transplantation and to reduce the risk of rejection across HLA-incompatibility. This entails identifying certain low-level DSAs (>3000 MFI) that may result in a positive flow cross match that are aggressively removed prior to transplantation via pheresis or immunoadsorption followed by targeted B and T-cell therapy to prevent further DSA formation.

Different combinations of therapies with varying amounts of success have been studied. IVIG therapy by itself has shown little promise [25] but IVIG in combination in rituximab alone [26] and rituximab and apheresis [27,28,29] or rituximab and immunoadsorption [30] have all been used successfully. Long term outcome studies have demonstrated a 5-year allograft survival as high as 80% in desensitized recipients of living donor kidney transplants [31]. Those who were desensitized had a clear survival benefit of 86% at 5 years [32] compared to 59.2% survival in those who remained on dialysis and 74.4% in patients who either waited or received a deceased donor kidney transplant during follow-up (*p* < 0.001). As such, desensitization can be a valuable tool to minimize rejection in highly sensitized individuals receiving HLA-incompatible transplants.

A wide variety of desensitization protocols have been employed by transplant centers depending on center-specific practices. Ranging from monthly IVIG for a total of 2 g/kg [33] to more aggressive therapy with 2 g/kg IVIG followed by 5–10 plasmapheresis treatments and 1–2 Rituximab doses [34].

Some studies have explored the use of novel agents for desensitization. Marks et al. examined the use of eculizumab versus standard of care (plasmapheresis +/− IVIG) and found no significant difference in treatment failure between the two groups at 9 weeks post transplantation (9.8% vs. 13.7%, *p* = 0.760). A French study looked at tocilizumab (IL-6 inhibitor) in comparison to standard therapy (apheresis and Rituximab) and found no difference in reduction of pre-transplant MFIs between the groups [35].

### 2.3. Role of Non-HLA Mismatches in Transplant Rejection

There is increasing evidence that donor-recipient mismatches at non-HLA regions may also be responsible for alloimmunity leading to chronic rejection and allograft loss. These mechanisms are discussed in detail by Jethwani et al. [36]. There are currently limited diagnostic and therapeutic options for the prevention or treatment of non-HLA antibody mediated rejection.

**Key Points of pre-transplant measures**

Decreasing the risk of sensitization by various methods.Better HLA matching includes eplet matching, especially in living donors.Use of densitization in highly sensitized recipients.



Now, we transition to the measures taken at and around the time of the transplant surgery.

## 3. Peri-Transplant Measures

### 3.1. Minimizing Cold-Ischemia Time/Optimizing Perfusion

A prolonged cold ischemia time of >24 h versus <12 h has been demonstrated to increase the rate of acute transplant rejection with relative risk of 1.13 (95% CI, 1.04–1.23) in first-time transplant recipients and relative risk of up to 1.66 (95% CI, 1.01–2.73) in retransplant candidates. This effect was not noted in recipients >60 years of age [37]. Strategies to minimize cold ischemia time may improve incidence of acute kidney transplant rejection rates, delayed graft function and allograft survival, especially in organs with a higher kidney donor profile index (KDPI) [38]. These strategies include simultaneous local and regional offers for higher KDPI kidneys and clear documentation by transplant centers about their organ acceptance criteria [38]. Several studies have shown improved rates of acute rejection with the use of hypothermic machine perfusion compared to static cold storage [39,40,41]. A meta-analysis comparing static cold storage to hypothermic machine perfusion did demonstrate improved DGF rates (RR 0.78, 95% CI 0.69–0.87, *p* < 0.0001), and improved graft survival at 3 years (RR 1.06, 95% CI 1.02–1.11, *p* = 0.009) but no difference in rate of acute rejection was identified [42]. Most transplant centers agree that this practice is useful to prolong the life of an allograft.

### 3.2. Individualizing Induction Immunosuppression

Induction immunosuppression is provided at the time of transplantation to minimize the risk of hyperacute and acute rejection. There are two categories of agents available: lymphocyte-depleting such as alemtuzumab (anti-CD52 antibody) and anti-thymocyte globulin (multi-cellular inhibition) and non-depleting such as basiliximab (IL-2 receptor antagonist) [Table 2].

Previous studies looking at induction vs. no induction agent demonstrated an overall reduction in deceased donor graft failure with lymphocyte-depleting agents [43] with reduction in allograft failure greater in patients with panel reactive antibody ≥ 20% (ratio of adjusted rate of 0.12, 95% confidence interval, 0.03–0.44; *p* = 0.002) [44]. It is now common practice to use induction immunosuppression at the time of transplantation. Choice of agent depends largely on anticipated immunologic risk. The definition of ‘high risk’ is controversial, heavily debated and factors in both recipient and donor characteristics. Studies comparing graft outcomes between basiliximab and anti-thymocyte globulin (ATG) defined high risk as black ethnicity, >3 HLA antigen mismatches, > 1 HLA-DR mismatch, higher PRA (>20%), cold ischemia time >24 h, delayed graft function, prior transplant and older donor age [45]. Compared to basiliximab, the anti-thymocyte globulin group had fewer incidences of acute rejection (15.6% vs. 25.5%, *p* = 0.02) and of acute rejection that required treatment with antibodies (1.4% vs. 8.0%, *p* = 0.005) [45]. However, it was noted that there was a significantly higher rate of infection in patients who received (ATG) compared with basiliximab. Interestingly, no studies have demonstrated a difference in patient or allograft survival between the two agents.

In most studies comparing alemtuzumab to ATG, there was no significant difference in biopsy-proven acute rejection or allograft survival [46,47,48] between the two agents suggesting no benefit of using alemtuzumab over anti-thymocyte globulin. A meta-analysis comparing induction with basiliximab to induction with ATG looked at pooled results from eight randomized controlled trials and found no significant differences in 1-year acute rejection rate (OR 1.32; 95% CI 0.93–1.87; *p* = 0.13), 1-year graft survival rate (OR 0.73; 95% CI 0.45–1.18; *p* = 0.20), 1-year patient survival rate (OR 0.52; 95% CI 0.27–1.02; *p* = 0.06) or 1-year infection rate [49]. However, the study did note a lower risk of neoplasm in the Basiliximab group (OR 0.26; CI 0.08–0.78; *p* = 0.02).

Another meta-analysis that compared efficacy and safety of induction agents found that alemtuzumab (OR 0.45, CI 0.29–0.78) and rATG (OR 0.63, CI 0.42–0.95) exhibited lower incidence of biopsy-proven acute rejection than basiliximab without a difference in graft survival. This came at the cost of a higher infection rate with ATG (OR, 1.8, CI, 1.01–2.8). [50]

In conclusion, use of ATG for induction is beneficial for individuals identified as having high risk immunologic profiles to minimize the risk of acute rejection. While comparison studies do not demonstrate convincing evidence of improved allograft survival using ATG vs. basiliximab, lack of long-term follow-up remains a limitation. In addition, use of ATG in high-risk individuals minimizes the cumulative burden of immunosuppression for treatment of acute rejection episodes. Infectious risk is an important factor that weighs into this decision due to greater infection rates with ATG use.

**Key points of peri-transplant measures**

Minimizing cold ischemia times and optimizing kidney perfusion are associated with a decreased risk of rejection.Induction immunosuppression should be individualized based on risk factors for rejection as highlighted and the risks of infections and malignancy in the long run.



We will now take a more detailed look into the post-transplant measures to reduce the risk of rejection in the kidney allograft.

## 4. Post-Transplant Measures to Prevent Rejection

The post-transplant phase is critical in the prevention of both acute and chronic rejection of the kidney allograft. This can be divided into the immediate post-operative period, in which maintenance immunosuppression is introduced into a recipient’s arsenal to prevent rejection and is a critical transition period where tolerability, therapeutic drug levels and early compliance is established. This is followed by the chronic post-transplant phase where compliance is cemented and a fine balance between immunosuppression and infection is maintained.

Conventional maintenance regimens consist of a combination of immunosuppressive agents that differ by mechanism of action and side effect profiles [Table 3]. This strategy minimizes the morbidity and mortality associated with each class of agent while maximizing overall effectiveness. Such regimens may vary by patient, transplant center and geographic area. [51]

The most common strategy is triple drug therapy with a combination of calcineurin inhibitor (CNI), antimetabolite (mycophenolic acid or azathioprine) and steroids. The use of non-CNI based regimens including co-stimulation blockers (Belatacept) and mTOR inhibitors (sirolimus or everolimus) is around 10%.

Serial discoveries and improvements in the drugs employed for maintenance immunosuppression have played a key role in the dramatic decrease in rates of acute rejection to <10% within the first year after transplant [52]. With excellent outcomes achieved in the short term, the aim of maintenance immunosuppression goes beyond prevention of acute rejection and includes the prevention of chronic allograft rejection and nephropathy [53].

This section will be sub-divided into the prevention of cell mediated and antibody mediated rejections.

### 4.1. Prevention of Cell Mediated Rejection

**T-cell proliferation and signaling pathways**

The key cellular mediator of rejection is the T-lymphocyte. Most immunosuppressive agents work by disrupting the keys pathways of T-cell activation. Signal 1 is initiated by the interaction of the T-cell receptor (TCR) on the T-cell with the Major Histocompatibility Complex (MHC) molecule on the Antigen Presenting Cell (APC) via the CD3 complex. Co-stimulation or Signal 2 constitutes the interaction of CD80 and CD86(B7) on the surface of APC and CD28 on T-cells. Both Signal 1 and 2 are essential to activate three signal transduction pathways: the calcium-calcineurin pathway, the RAS-mitogen activated protein (MAP) kinase pathway and the nuclear factor-kb pathway [54]. These pathways activate transcription factors that trigger the expression of many new molecules, including interlekin-2 (IL-2), CD154 and CD25. IL-2 and other cytokines activate the “target of rapamycin” pathway to provide Signal 3, the trigger for cell proliferation. Nucleotide synthesis is also required for lymphocyte proliferation and the mobilization of effector T-cells. Different classes of immunosuppressive agents target different steps of the T-cell proliferation pathways to prevent their activation and subsequent rejection of the allograft. These mechanisms have been explained in more detail in another article in this series titled *Pathophysiology of rejection in kidney transplantation.*

CNIs are considered the backbone of maintenance immunosuppression and are used by a vast majority of transplant centers in the United States [52] Cyclosporine and tacrolimus are the two agents of this class in clinical use and voclosporin remains an investigational agent currently [55]. CNIs bind to their binding proteins (FKBP for tacrolimus and cyclophilin for cyclosporine) and inhibit calcineurin. This inhibition blocks the dephosphorylation and activation of nuclear factor NFAT thus preventing transcription of IL-2 which is critical to lymphocyte proliferation. Cyclosporine was touted as a gamechanger after its discovery in the 1980s. However, it has largely been replaced by tacrolimus as the CNI of choice in most immunosuppression regimens. A large meta-analysis of 30 trials (4102 patients) comparing these two agents favored tacrolimus for multiple endpoints with a 44% reduction in death-censored graft failure and 31% reduction in the risk of acute rejection within 1 year of transplant [56]. This analysis also revealed a significantly higher risk of development of insulin-dependent diabetes, neurological and GI side effects with tacrolimus. Given the side effect profile and varying sensitivity to the two drugs, an individualized approach in deciding a CNI may be needed in certain patients.

Antimetabolites are an integral part of maintenance immunosuppression regimens. The most used agents are Mycophenolic acid (MPA) and azathioprine. These agents inhibit nucleotide synthesis which limits T and B-lymphocyte proliferation. MPA was approved by the FDA for the prevention of rejection in 1995. This was based on the Tricontinental Study (North America, Europe and Australia) which showed a significantly lower risk of acute rejection in kidney transplant recipients on MPA compared to Azathioprine [57]. A meta-analysis of 23 studies which included 3301 participants showed the MPA was superior to azathioprine in terms of the risk of graft loss including death (RR 0.82), death-censored graft loss (RR 0.78) and any acute rejection (RR 0.65) [58]. Thus, MPA has become the favored agent in combination with CNIs in most patients for the prevention of acute rejection. It is contra-indicated in pregnancy as it is teratogenic.

#### CNI Free Regimens

Although tacrolimus remains the main component of maintenance immunosuppression, it is associated with significant toxicities and thus may not be usable in all patients. In such situations, CNI free regimens have been established. Belatacept is a first in class co-stimulation blocker which interacts with Signal 2 and thus selectively blocks T-cell activation. It was approved by FDA in 2011 based on two landmark trials which compared Belatacept with cyclosporine for maintenance immunosuppression both in standard and extended criteria kidneys. Although the rate of acute rejection was higher in the Belatacept cohorts, however, this did not have an impact on long term patient and graft survival [59,60]. Subsequent meta-analysis of 5 studies that compared Belatacept and CNIs (1535 patients) have reported similar rates of death, allograft survival and acute rejection after 3 years of transplant [61]. Studies of conversion from CNI to Belatacept have also shown similar trends [62]. mTOR inhibitors engage FKBP12 to create complexes that inhibit the target of rapamycin which blocks Signal 3 by preventing cytokine receptors from activating the cell cycle. mTOR inhibitors have been evaluated in several regimens but have not shown to be superior to either CNIs or antimetabolites in prevention of rejection. However, they do have anti-viral and anti-tumor activity and thus are favored in such situations [63].

Corticosteroids remain an integral part of most immunosuppressive regimens. They inhibit production of activating cytokines and downregulate the expression of activating molecules on the surface of T-lymphocytes. However, recent trends have seen an increase in steroid free regimens to minimize long term side effects [52].

### 4.2. Prevention of Antibody Mediated Rejection

Antibody-mediated rejection (AMR) is a significant complication following kidney transplantation that contributes toward both short- and long-term injury in approximately 1% to 10% of kidney transplant recipients [64]. Certain factors including allo-sensitization, patient non-compliance and iatrogenic reduction in immunosuppression contribute significantly to the emergence of de novo HLA and non-HLA antibodies or persistence of pre-existing antibodies. This increases risk for chronic AMR which is thought to be a significant cause of premature graft failure [65,66,67]. The best treatment for AMR is to prevent it. Post-transplant prevention of AMR should involve a multipronged strategy.

Maintenance of immunosuppression remains a key element in this approach. Therapeutic tacrolimus levels are associated with reduced production of de novo DSA [68]. CNI minimization strategies have been proposed and used to mitigate the potential side effects of CNIs including nephrotoxicity which was thought to contribute significantly to late allograft loss. However, given the extensive research to suggest that chronic AMR is the bigger culprit [65,66,67], care providers must be careful when making changes to immunosuppression regimens, including lowering targets for CNI troughs and weighing the potential risks/benefits, especially in highly sensitized transplant patients. Mycophenolate Mofetil (MMF) is associated with decreased formation of de novo DSA and rates of biopsy proven AMR as compared to no MMF/Azathioprine in combination with a CNI [58,69]. Belatacept has also been shown to lower the incidence of donor specific antibody (DSA) formation both when used de novo and when used as a CNI conversion strategy [59,60,70]. Belatacept-based immunosuppression decreases pre-existing antibodies [71]. Thus, careful curation of immunosuppression strategies considering demographic variables, immunological risk and patient preferences should be made to minimize the risk of AMR and prevention of premature graft loss.

Another aspect of this multipronged strategy involves monitoring for the development of DSA. Currently there are no standard guidelines for this approach, and individual centers create their own protocols based on multiple factors including patient variables, HLA lab and logistical support and cost. It is debatable if lab monitoring for alloantibodies is needed in all transplant patients, but it seems justified in immunologically high-risk patients, desensitized recipients, patients with a suspicion for rejection and during treatment of an AMR to recognize allograft injury early and prevent its translation into chronic rejection [72].

### 4.3. Minimizing Non-Compliance

Non-compliance is a major risk factor for rejection and has been associated with premature graft loss [73,74]. It encompasses multiple aspects of transplant related care including immunosuppression medication, lab monitoring, transportation to medical visits and lifestyle modifications to minimize the risk of non-immunological graft injury and subsequent failure. Certain demographic groups are considered high risk including teenagers and young adults who are transitioning from the pediatric to adult renal services, certain social and ethnic groups and individuals known to be facing financial crises that make drugs and ongoing medical care unaffordable [74]. Given the seriousness and potential graft threatening consequences of non-adherence, every effort should be made to address this early as the likely benefits of this intervention will be diminished after emergence of de novo DSA [75]. Enhanced surveillance, especially for higher risk groups, may include interviews including additional use of telemedicine, visits from social workers, utilizing the transplant pharmacy teams to monitor prescription filling records and reiterating medication education and additional antibody screening at regular intervals especially for higher immunological risk patients.

### 4.4. Treatment of Rejection

Patients at risk for denovo DSA formation have preceding cellular rejections with more intense inflammation within the microvasculature and if not treated early and aggressively, it is postulated that peritubular capillaritis can lead to increased HLA expression in the microcirculation, thereby increasing the risk of allo-recognition by the recipient B-cell compartment. Moreover, when cellular rejection coincides with DSA and antibody-mediated microvascular injury, it may accelerate the time to graft dysfunction and graft loss [73]. This underscores the importance of aggressive treatment for active cellular or antibody mediated rejection to decrease the risk of chronic rejection and graft loss [73]. After addressing the acute inflammatory component with appropriate intervention, the treating physician should make sure to optimize the maintenance immunosuppression. This may include resetting target troughs for CNIs, especially if a CNI minimization strategy has been previously employed for the patient, re-introduction or increase in antimetabolite or consideration of switch to Belatacept if CNI avoidance is warranted. Treatment strategies, including details of medications used, for the same are outlined in another article of this series titled *Current therapies in Kidney Transplant Rejection*.

### 4.5. Role of Biomarkers

A multitude of novel biomarkers have been developed to assess allograft health and predict rejection before changes in GFR take place and to predict long term graft outcomes. Only a few are currently available in clinic practice. A potential early indicator for the injury and loss of allograft is donor derived cell free DNA circulating in the blood of transplanted patients. This is measured as a percent of recipient circulating DNA and increase in this fraction is a sensitive marker of allograft injury [76]. Three assays, based on NGS technology: Allosure, TRAC and Prospera are currently available for commercial use. These have been validated and are most useful for detection of AMR [77,78]. Gene expression profiling based non-invasive test available as TRUGRAF is validated for detecting subclinical rejection [79]. These tests can potentially be used for monitoring allograft function post rejection treatment and can direct immunosuppression therapy changes to balance the risk of future rejection and adverse effects. One major hindrance to their consistent use is the associated cost and thus the companies offering these tests should work towards subsidizing them over time. Other promising biomarker classes which can potentially contribute to the prevention of both acute and chronic rejection include chemokines, free microRNAs and leucocyte subclasses [80].

### 4.6. Emerging Therapies

Given the significant improvements in acute cellular rejection rates over time [52], there have not been any major efforts to make newer drugs affecting T-lymphocyte signaling. Novartis launched the CIRRUS-1 study in 2018 to evaluate the safety and efficacy of iscalimab (a non-B-lymphocyte depleting anti CD-40 monoclonal antibody) in kidney transplant recipients [81]. However, this was terminated early due to an interim analysis showing inferiority to tacrolimus-based regimens for rejection prevention. Clazakizumab, a monoclonal antibody against the IL-6 ligand has shown promising results in a phase 2 pilot RCT [82]. It decreased the DSA levels in patients with AMR after 1 year of transplant and showed a significantly slower decline in GFR as compared to placebo. A large phase 3 trial of clazakizumab in patients with chronic active AMR is currently ongoing [83]. Tocilizumab, a monoclonal antibody against the IL-6 receptor has shown promise both in desensitization protocols as well as for treatment of chronic AMR [84,85]. Larger RCTs are needed to confirm these findings and justify the use given significant cost associated with this drug.

Daratumumab is a monoclonal antibody against CD38 which has shown promising results in decreasing anti-HLA antibodies in AMR [86,87] and has been proposed as an agent for desensitization and treatment of AMR. However, more concrete evidence is needed before its acceptance in clinical practice.

Belimumab is a humanized, monoclonal, anti B-lymphocyte stimulator (BLyS) IgG1 antibody that prevents B-cell survival and differentiation into plasma cells. In a RCT with 28 kidney transplant recipients, there was no significant difference in the risk of major infections compared to standard immunosuppression but the IL-10/IL6 ratio of the B-cell distribution was skewed towards a regulatory profile and activated memory B-cells and plasmablasts were significantly reduced [88] This likely has the downstream effect of decreasing the risk of rejection. However, specific studies are needed to address this.

Imlifidase, an IgG degrading enzyme of Streptococcus pyogenes (IdeS), cleaves human IgG at a specific amino acid sequence within the hinge region producing Fc and F(ab)s fragments effectively blocking complement dependent cytotoxicity and antibody dependent cellular cytotoxicity [89]. It was associated with rapid reduction and even elimination of DSA [90,91]. However, rebound in DSA and anti-IdeS antibody development are significant issues associated with its use. This, along with the patient population that it will benefit the most will need to be addressed in future studies.

Berinert and Cinryze are plasma C1-esterase inhibitors that have been tested in two pilot studies and have shown a functional improvement in AMR [92,93]. More trials are currently underway and hopefully will define the role of these agents in the prevention of AMR.

A more detailed discussion on these agents is covered in a subsequent article of the series.

**Key points of the post-transplant measures**

Maintenance immunosuppression is a key part of preventing acute and chronic rejection and should be individualized and offer different mechanisms and less side effects.Strategies should be devised to increase long-term compliance.Acute rejections should be treated aggressively to decrease risk of chronic rejection.Novel biomarkers should be utilized in a judicious manner.Emerging therapies offer promise but need more studies.



## 5. Financial Impact, Practical Considerations and Real-World Challenges

Kidney transplantation is considered the best modality to manage ESRD. It has not only shown improved outcomes, both in terms of mortality and quality of life as compared to dialysis, but multiple studies have shown its cost-effectiveness and economic advantages over dialysis [94,95]. While there are certain sub-groups which incur a higher cost initially, in the long run kidney transplantation is consistently considered the superior financial option. Thus, every effort should be made to improve long-term graft outcomes and preventing rejection is a key component of the same. There are real-world challenges and practical considerations as it relates to maintaining compliance over long periods of time, including insurance coverage for medications and clinical care, logistical challenges related to transportation and an ever-increasing complexity of transplant patients which warrants a steady increase in the personnel to manage them effectively.

## 6. Conclusions

**Figure 1:**
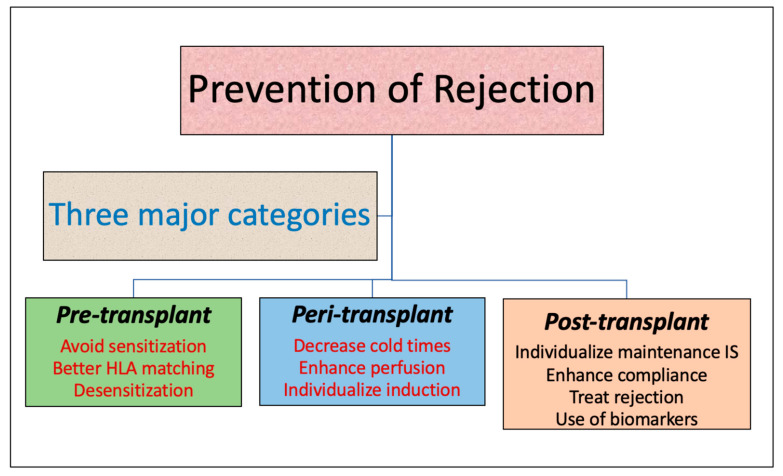
Prevention of rejection.

**Figure 2:**
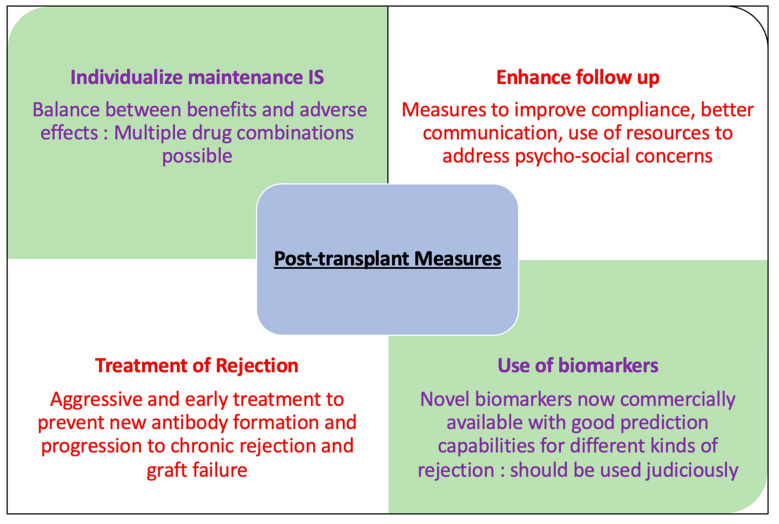
Post-transplant measures to prevent rejection.

Prevention of rejection is key for the longevity of kidney allografts. This can be achieved at various stages in the lifetime of the graft and requires a multipronged approach and significant effort on the part of the recipients and transplant centers [Figure 1 and Figure 2]. We recognize the limitations of the current research, especially the lack of long-term follow-up for many studies of induction and maintenance immunosuppression therapy. Emerging therapies are yet to prove their worth in RCTs and long-term studies. Future research should focus on long-term graft outcomes and highlighting the comparative performance of common induction and maintenance agents as well as development and validation of newer therapies for rejection prevention. There is a lot of interest in the novel biomarkers as tools for immunosuppression modulation and prediction of allograft injury much before the usual markers of graft function like serum creatinine, urine protein, etc. are affected. However, these novel biomarkers are expensive and present significant logistical challenges including long turn-around times. Thus, a significant focus needs to be directed towards these issues.

## Figures and Tables

**Table 1:** Prevention of rejection in kidney allograft.

***Pre-transplant***
Avoiding sensitization
HLA matching including eplet matching
Desensitization
***Peri-transplant***
Minimizing cold ischemia time/Optimizing perfusion
Individualizing induction immunosuppression
HLA matching including eplet matching
***Post-transplant***
Individualizing maintenance immunosuppression
Minimizing non-compliance
DSA monitoring
Treatment of acute rejection
Role of novel biomarkers

**Table 2:** Induction Immunosuppression Agents.

Agent	Class	Mechanism of Action	Adverse Effects
Basiliximab	Lymphocyte Non-depleting	Blocks IL-2 receptor	Minimal
Rabbit anti-thymocyte globulin	Lymphocyte depleting	Polyclonal rabbit antisera; destroys T lymphocytes by targeting multiple antigens	Infections, Bone marrow suppression, infusion reactions
Alemtuzumab	Lymphocyte depleting	Humanized rat monoclonal antibody; destroys T lymphocytes and other immune cells by targeting CD-52	Infections, Bone marrow suppression

**Table 3:** Maintenance Immunosuppression Agents.

Drug	Class/Mechanism	Use	Adverse Effects	Practical Considerations
Tacrolimus	CNI	Used in a vast majority of combination regimens for maintenance immunosuppression	Neurotoxicity which includes tremor, AMS and memory loss, nephrotoxicity, increased incidence of DM, Hair loss	Component of >90% regimens in the US. Also available as a long-acting formulation called Envarsus
Cyclosporine	CNI	Limited use in current regimens for maintenance immunosuppression	Hirsutism, gingival enlargement, nephrotoxicity	Was approved more than a decade before tacrolimus but is now primarily used in patients who are intolerant of tacrolimus
Mycophenolic Acid	Antimetabolite	In combination with tacrolimus makes up more than 90% of maintenance immunosuppression regimens	GI side effects including nausea, vomiting and diarrhea; bone marrow suppression	Contraindicated in pregnancy
Azathioprine	Antimetabolite	Used in combination with CNI/Belatacept/mTORi rarely	Bone marrow suppression, hepatotoxicity	Useful in pregnancy and in case of intolerable side effects to MPA. Genetic polymorphisms exist that alter metabolism
Prednisone	Corticosteroid	Used in about 70% of maintenance immunosuppression regimens	Metabolic side effects, osteoporosis, weight gain	Can be used as part of induction, maintenance, and rejection treatment regimens
Sirolimus/ Everolimus	mTOR inhibitor	Used rarely either in combination with CNI or other agents	Hyperlipidemia, lymphedema, bone marrow suppression, lung injury, oral ulcers, proteinuria, impaired wound healing	Useful in special circumstances like skin cancer and certain viral infections like BK and CMV
Belatacept	CTLA-4 Ig fusion; works as co-stimulation blockade to selectively halt T-cell activation	Used as part of maintenance immunosuppression in patients who cannot tolerate CNI; de novo use is rare	Infusion reactions, bone marrow suppression, diarrhea	Black box warning in EBV-seronegative patients: increased risk of PTLD. Use is increasing because of less nephrotoxicity. Cost remains a major issue

## Data Availability

No new data were created or analyzed in this study. Data sharing is not applicable to this article.
